# Editorial: Advancing the representation of minoritized groups and social determinants of health in brain injury research

**DOI:** 10.3389/fneur.2023.1169445

**Published:** 2023-04-11

**Authors:** Kelli W. Gary, Jessica S. Wallace, Rebekah Mannix, Monique R. Pappadis

**Affiliations:** ^1^Department of Rehabilitation Counseling, Virginia Commonwealth University, Richmond, VA, United States; ^2^Department of Health Science, The University of Alabama, Tuscaloosa, AL, United States; ^3^Division of Emergency Medicine, Department of Pediatrics, Boston Children's Hospital, Boston, MA, United States; ^4^Department of Pediatrics, Harvard Medical School, Boston, MA, United States; ^5^Department of Emergency Medicine, Harvard Medical School, Boston, MA, United States; ^6^Department of Population Health and Health Disparities, School of Public and Population Health, and Sealy Center on Aging, University of Texas Medical Branch (UTMB) at Galveston, Galveston, TX, United States; ^7^Brain Injury Research Center, TIRR Memorial Hermann Hospital, Houston, TX, United States

**Keywords:** traumatic brain injury, social determinants of health, minoritization, epidemiology, acquired brain injury

Acquired brain injury (ABI), including traumatic brain injury (TBI) and stroke, is a global public health concern, with an estimated 43.6 million new cases in 2016 (1). Unfortunately, large gaps in healthcare and support across the continuum of care persist for persons with ABI, often exacerbated by social determinants of health (SDoH). As indicated by Healthy People 2030, SDoH include individual- and system-level factors that influence “environments where people are born, live, learn, work, play, worship, and age that affect a wide range of health, functioning, and quality-of-life outcomes.” The domains of SDoH are economic stability, education access and quality, health care access and quality, neighborhood and built environment, and social and community context (2). Yet, despite increased understanding of the effects of SDoH on health outcomes, less is known about its role in health and health outcomes amongst minoritized groups with ABI. Thus, the call by Frontiers in Neurology, Neuroepidemiology to address SDoH in ABI is relevant and timely.

This Research Topic, promoting intentional research for and with minoritized groups in the US and abroad, includes several articles in the brain injury field about the outcomes and contributing factors for historically marginalized populations, such as racial/ethnic minorities, sex and gender minorities, survivors of domestic violence, and people who experience incarceration, among others. Guest editors of this Research Topic have reported on perceptions of recovery following TBI for Spanish-speaking US immigrants (3), racial and ethnic differences in employment and other post-injury outcomes after TBI (4, 5), mobility and post-hospitalization outcomes for Mexican American Medicare beneficiaries (6), racial disparities in diagnosis of concussion and minor head trauma in emergency department visits for adults and pediatric patients [Lempke et al.; (7)], and race/ethnicity and retention in TBI outcomes research (8). Although significant research covers specific domains of SDoH in brain injury (9–11), there is a dearth of evidence addressing both SDoH and minoritized populations. More brain injury research with an explicit focus on SDoH will lead to better holistic interventions and best practice strategies in this area.

This Research Topic of studies explores SDoH factors for adult, adolescent, and pediatric patients in the US, Canada, Sub-Saharan African nations, and Ecuador. Further, these studies contribute empirical evidence in brain injury research to identify gaps in the current literature to inform novel interventions for minoritized groups. Half of the studies in our Research Topic focus on individuals in the continental US. Garduño-Ortega et al. explore the compounding effect of membership in eight adult marginalized groups (racial and ethnic minority status, low education status, low English proficiency, abuse of substances, homelessness, psychiatric hospitalizations, psychiatric disorders, and incarceration history) on outcomes 1-year post-TBI. They found that membership in four or more marginalization groups had a compounding negative effect on TBI severity, recovery, and employment. Hudac et al. investigated the association between cognitive inhibition and frustration induction using high-density mobile electroencephalography (EEG) among Black adolescent athletes with concussion. EEG was sensitive to frustration induction during preseason, but was less effective for athletes with a history of concussion. Wells et al. examined several social determinants as predictors of consent to participate and be retained in a prospective longitudinal cohort study of pediatric patients with mild TBI (mTBI) or orthopedic injury. Although consent rates were higher in Black and multi-racial children, participants who were multi-racial or white were more likely to be retained in a 2-week post-acute assessment, including magnetic resonance imaging. No follow-up difference by race was identified among children retained in the study. Finally, Lempke et al. used 6 years of emergency department data from the National Hospital Ambulatory Medical Care Survey to determine the association between race/ethnicity and emergency department visits for concussions, their mechanism of injury, and computed tomography (CT) scan use. Among adult (age 18–45) visits examined, no association existed between race/ethnicity and visits that resulted in a concussion diagnosis, after controlling for sex and primary insurance type; however, there were racial/ethnic differences in the underlying mechanism for concussion and Hispanic patients had lower odds of receiving a CT scan than non-Hispanic white patients.

Other studies provide an international perspective. Baiden et al. found that 3.3% of the patients in the Kintampo Injury Registry in Ghana had a head injury. Males had 1.4 times greater odds than females of sustaining a head injury. Anto-Ocrah et al. examined the intersection between intimate partner violence (IPV) and TBI in Sub-Saharan Africa, where IPV is particularly high. This scoping review of 10 articles included studies from Uganda, Ghana, South Africa, and Cameroon, demonstrating that both subjective and objective assessments of IPV and TBI are possible in more resource-limited settings. This piece also highlights the clinical importance of including gender as a social determinant of brain health. Carrington et al., in a retrospective analysis of a publicly-available national health dataset in Ecuador, found that stroke incidence was higher in males, but females had a higher death rate. The highest stroke incidence was among the Mestizo ethnic group; the Montubio ethnic group had the highest fatality rate. Finally, Chan et al., in Toronto, Canada, completed a scoping review of the availability and extent of rehabilitation for individuals with TBI who intersect with the criminal justice system. The 22 peer-reviewed articles and two gray literature reports that met eligibility criteria found existing rehabilitation interventions being used; opportunities to integrate TBI rehabilitation interventions with the criminal justice system; and gaps in knowledge reports by sex, gender, and other social identities.

The studies in this Research Topic highlight brain injury across the lifespan and significantly contribute to the literature by engaging and including persons with ABI from historically marginalized populations. There is substantial heterogeneity among findings in this Research Topic. For example, there are significant differences in findings noted by Garduño-Ortega et al. for urban adults with TBI where more marginalization had a compounding negative effect on injury severity, recovery, and post-injury competitive employment and Lempke et al. who found no association between race/ethnicity and concussion after accounting for covariates. Chan et al. noted that there was minimal information about race and ethnicity in a majority of articles included in scoping review about CJS but they recognized the importance of considering sex, gender, and other intersecting factors in this population. Whereas, Wells et al. found differences in recruitment and retention with marginalized groups faring better than nonmariginalized groups, but were less represented in post-acute visits. Hudac et al. had implications for community engaged methods to increase representation of marginalized groups. Urban vs. other environmental settings, adults vs. adolescents and children, and ED vs. post-acute settings may account for the varying results. Additionally, SDoH are complex and multidimensional factors; therefore, it is imperative to interpret these results from multiple perspectives. This also holds true for the interactional studies with very different background and data surveillance. Collection of epidemiological, cultural, and community-engaged work not only expands the literature but also highlights the need for scrupulous and purposeful research in this area. Together, these studies demonstrate structural inequities in who sustains an ABI, who gets diagnosed with one, who gets treated for one, who gets included (and retained) in ABI studies, and even who researches ABI. They represent a call to action to improve health outcomes in all those with ABI.

## Author contributions

KG, JW, RM, and MP contributed to the conceptual development of the editorial and the drafting of the editorial and agreed to the final draft of the editorial. All authors contributed to the article and approved the submitted version.
